# The impact of gender difference on operative time in laparoscopic partial nephrectomy for T1 renal tumor and the utility of retroperitoneal fat thickness as a predictor of operative time

**DOI:** 10.1186/s12885-016-2979-5

**Published:** 2016-12-12

**Authors:** Hiroki Ito, Kazuhide Makiyama, Takashi Kawahara, Kimito Osaka, Koji Izumi, Yumiko Yokomizo, Noboru Nakaigawa, Masahiro Yao

**Affiliations:** Department of Urology, Yokohama City University Graduate School of Medicine, 3-9, Fukuura, Kanazawa-ku, Yokohama, Kanagawa Japan

**Keywords:** Renal cancer, Laparoscopy, Partial nephrectomy, Gender difference

## Abstract

**Background:**

To investigate the impact of biological gender on operative parameters, especially operative time, in laparoscopic partial nephrectomy (LPN) for T1 renal tumor.

**Methods:**

One hundred and eleven (28 female and 83 male) patients and 64 (20 female and 44 male) patients with renal tumors suspected to be RCC cT1aN0M0 who underwent retroperitoneal and transperitoneal LPN, respectively, were analyzed. The influence of sex on operative factors including retroperitoneal fat tissue thickness, determined on CT, was analyzed. The correlation between operative time and gender was evaluated by unpaired t-test and linear logistic regression model.

**Results:**

In both retroperitoneal and transperitoneal LPN, the retroperitoneal fat tissue thickness was greater in men than in women. In retroperitoneal LPN, the operative time was significantly longer in men than in women. In contrast, in transperitoneal LPN, no gender difference was observed in regard to the operative time. In retroperitoneal LPN, linear logistic regression assessment showed that gender, retroperitoneal fat tissue thickness, and tumor size were significantly associated with operative time. Coefficient of determination of the prediction model was 0.317.

**Conclusions:**

The operative time of retroperitoneal LPN is significantly correlated with gender, maximum tumor diameter, and retroperitoneal fat tissue thickness. We have developed a prediction model for the operative time of retroperitoneal LPN based on preoperative parameters. Interestingly, in transperitoneal LPN, a gender difference in operative time was not apparent, and also predicting operative time might be difficult.

## Background

In the last two decades, partial nephrectomy (PN) has emerged as a treatment that is oncologically equivalent to radical nephrectomy in most cases of localized renal cell carcinoma (RCC) in terms of cancer-specific survival and overall survival [1, 2]. The use of PN for the treatment of small renal masses has increased over time and is now a recommended, standard treatment for all clinical stage I renal masses [3].

There have been discussions on the influence of biological gender on the treatment of RCC. Some previous reports indicated that there is gender difference in the surgical management of the small renal mass, and they all indicated that women are less likely to undergo PN compared with men, though the exact reason for this gender discrepancy is not known [4–6]. In addition, gender medicine research has outlined the impact of gender on physiology and pathology of diseases. Several studies addressed the impact of gender on RCC pathology [7–10], RCC survival [7–10], and benign renal masses [11]. Multinational mate-analysis study demonstrated that men present with larger, higher stage, higher grade RCC than women and overall survival is better in women, whereas cancer-specific survival is not significantly different [7].

These previous studies clearly indicated that a gender difference is observed in the pathological and clinical findings of RCC, but gender was not found to play a role in patient selection for surgical treatment so far. We had the clinical impression that the operative time of laparoscopic PN (LPN) took longer in male than female patients because the management of fat tissue surrounding the kidney seems to be more difficult in male patients than in female patients. Currently, we think that there is a need to evaluate the relationship between gender and the clinical operative parameters of LPN. The present study aimed to investigate the impact of gender on operative parameters, especially operative time, in laparoscopic PN (LPN) for T1 renal tumors.

## Methods

### Patients

One hundred and seventy-five consecutive patients with renal tumors that were suspected to be RCC cT1aN0M0 who underwent LPN at Yokohama City University Hospital between May 2003 and September 2015 were retrospectively reviewed. Among them, 111 (28 female and 83 male) patients and 64 (20 female and 44 male) patients who underwent a retroperitoneal and transperitoneal LPN, respectively, were analyzed in this study. All procedures were performed with the arterial clamping method by a single surgeon (M.K.).

According to our hospital’s criteria for selecting the surgical method for RCC, the main indication for LPN (both retroperitoneal and transperitoneal approaches) is RCC cT1N0M0. The choice of approach is based on the tumor location. Among LPN cases, the retroperitoneal approach was chosen for tumors located on the posterior side of the kidney, while the transperitoneal approach was chosen for all other tumors. For cT2-T3aN0M0 RCC, as well as cT1 tumors located at the central part of the kidney, open PN or open/laparoscopic radical nephrectomy was indicated. In our institution, all of final pathological diagnosis were done by expert pathologists (more than 10 years experienced) on the basis of valid WHO classification at the moment of diagnosis.

This study was approved by the ethics committee of Yokohama City University Hospital. Written informed consent was obtained from all patients for their data to be used for research purposes.

### Surgical techniques

The details of the surgical techniques in our hospital have been previously reported in detail [12, 13]. Briefly, after Gerota’s fascia is opened, the renal capsule is visualized around the tumor. After visualization with ultrasound, the renal capsule is cut in a monopolar fashion around the tumor. After the renal artery is clamped with a bulldog clamp, cold cutting by scissors into the renal parenchymal boundary of the tumor is performed with an optimal surgical margin (a few millimeters). After retrograde injection of diluted indigo carmine, continuous suturing of the opened collecting system and transection of the major vessels is performed with intracorporeal knot-tying. Parenchymal suturing is performed in a continuous fashion. The 20–30 cm length of thread is used, and a knot is made at the end of the thread. A large Hem-o-lok polymer clip (Weck Closure System, Research Triangle Park, NC) is attached on the proximal side of the knot. Before the thread is tightened or cinched, the parenchyma is sutured in a running fashion with three or four stitches without any bolster so that the renal bed is kept in its natural position during the suturing. The thread is tightened from the distal to proximal end with application of suitable tension. Subsequently, the tightened thread is fixed with a large Hem-o-lok, one stitch at a time.

### Clinical parameters

**Fig. 1:**
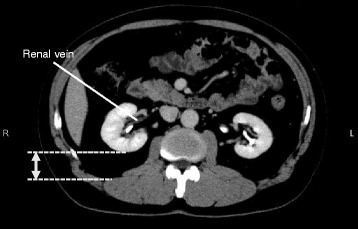
The methodology of measurement of retroperitoneal fat tissue thickness using axial computed tomography imaging. The measurement of retroperitoneal fat tissue thickness is performed at the slice that showed the renal vein on the treated kidney side

The clinical factors analyzed in this study included the gender of the patients, operative time, maximum tumor diameter, laparoscopic approach (retroperitoneal or transperitoneal), retroperitoneal fat tissue thickness, R.E.N.A.L. Nephrometry Score [14], volume of bleeding, weight of the specimen, and postoperative pathological findings, including the histologic subtypes. The retroperitoneal fat tissue thickness, the R.E.N.A.L. Nephrometry Score and the tumor diameter were measured by a urologist (H.I.) using preoperative CT scans. The measurement of retroperitoneal fat tissue thickness is straightforward, using the axial CT image at the renal vein level of the treated kidney (Fig. 1).

### Statistical analysis

Statistical analysis was performed using the Statistical Package for Social Sciences, version 23 (SPSS Inc., Chicago, IL). Gender difference in patient characteristics and preoperative factors was analyzed using the unpaired *t*-test and chi-square test.

Pearson’s coefficient was used to measure the correlation between the operative time and retroperitoneal fat tissue thickness. Linear logistic regression models were used to assess the potential predictive factors for operative time of LPN. The correlation between the operative time of LPN and gender was also evaluated by the unpaired *t*-test.

## Results

### Gender difference in patients’ background characteristics and operative parameters

**Fig. 2:**
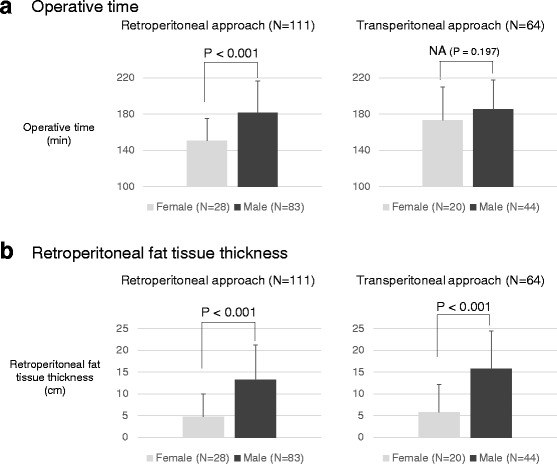
The bar chart indicated the sex differences in the operative time (**a**) and retroperitoneal fat tissue thickness (**b**) in the two procedures, the retroperitoneal approach and transperitoneal approach

The patients’ background characteristics, tumor factors, and operative factors in the patient groups who underwent retroperitoneal and transperitoneal LPN are shown in Table 1. The comparison of the operative time and retroperitoneal fat tissue thickness between men and women are shown in Figs. 2a and b.

**Table 1:** Comparison of patient characteristics and pre- and perioperative factors between genders

A. Retrospective approach (*N* = 111)			Female (*N* = 28)	Male (*N* = 83)	*P* value
Age (years)			55.5 ± 12.5	58.9 ± 12.8	0.226
Side (no.)	Right/Left		15/13	33/50	0.202
Height (cm)			156.2 ± 6.9	168.6 ± 7.2	<0.001^a^
Body weight (kg)			53.4 ± 9.4	69.8 ± 13.9	<0.001^a^
Body mass index (cm/kg2)			22.0 ± 3.7	24.4 ± 3.8	0.005^a^
Max. tumor diameter (mm)			23.3 ± 6.7	24.1 ± 7.3	0.605
The R.E.N.A.L. Nephrometry Score			5.7 ± 1.5	5.8 ± 1.4	0.954
Ischemia time			14.6 ± 9.0	19.2 ± 11.9	0.069
Resected volume (g)			7.3 ± 2.9	14.9 ± 14.7	0.018^a^
Histological findings	histologic subtypes	clear cell	12	62	<0.001^a^
		papillary	0	8	
		chromophobe	5	1	
		oncocytoma	0	5	
		angiomyolipoma	6	2	
		other	5	5	
Blood loss (ml)			16.9 ± 23.8	109.5 ± 247.8	0.056
Surgical complications	Peritoneal injury		3	4	0.395
B. Transperitoneal approach (*N* = 64)			Female (*N* = 20)	Male (*N* = 44)	*P* value
Age (years)			56.6 ± 16.8	62.3 ± 11.8	0.122
Side (no.)	Right/left		12/8	30/14	0.523
Height (cm)			155.2 ± 6.7	168.0 ± 6.0	<0.001^a^
Body weight (kg)			55.1 ± 9.1	70.2 ± 10.7	<0.001^a^
Body mass index (cm/kg2)			22.9 ± 4.0	24.9 ± 3.6	0.057
Max. tumor diameter (mm)			22.4 ± 8.6	25.8 ± 8.2	0.149
The R.E.N.A.L. Nephrometry Score			6.0 ± 1.5	6.6 ± 1.4	0.106
Ischemia time			18.7 ± 9.6	18.4 ± 8.8	0.924
Resected volume (g)			9.7 ± 6.3	15.8 ± 11.2	0.019
Histological findings	histologic subtypes	clear cell	12	32	0.413
		papillary	1	4	
		chromophobe	1	2	
		oncocytoma	1	1	
		angiomyolipoma	3	1	
		other	2	4	
Blood loss (ml)			86.5 ± 94.4	60.6 ± 89.0	0.306
Surgical complications	Ileum injury		0	1	NA

^a^Significant differences between the two groups as determined by unpaired t-test, Mann-Whitney’s U test or chi-square test

In both retroperitoneal and transperitoneal LPN, the retroperitoneal fat tissue thickness (*P* < 0.001 in both retroperitoneal and transperitoneal LPN) and resected tumor volume (*P* = 0.018 and *P* = 0.019, respectively) were higher in men than in women. On the other hand, in both approaches for LPN, there was no significant gender difference in maximum tumor diameter, R.E.N.A.L. nephrometry score, or ischemia time.

In retroperitoneal LPN, the operative time (*P* < 0.001) was significantly longer in men (181.8 ± 34.9 min) than in women (150.7 ± 24.7 min). On the other hand, in the transperitoneal LPN, no difference was observed in the operative time between men (185.6 ± 32.2 min) and women (173.0 ± 36.8 min).

### Intraoperative complications

In terms of surgical complications, an ileal injury caused by trocar insertion was observed in one case of transperitoneal LPN (Table 1) and treated by suturing the injured site during surgery and subsequent antibiotic treatment.

### Correlation between the operative time and retroperitoneal fat tissue thickness

**Fig. 3:**
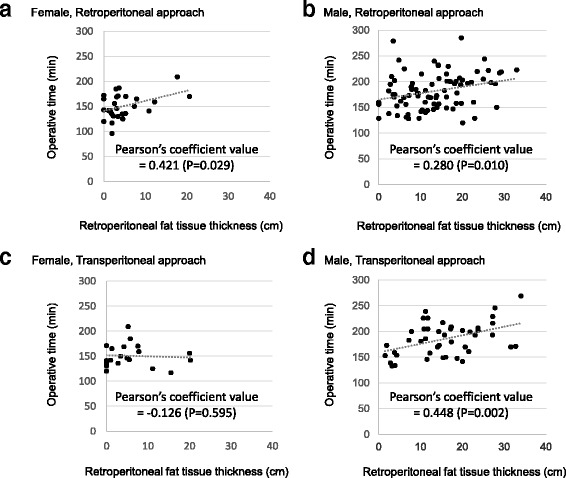
The scatter plot graph indicates the correlation between the operative time and retroperitoneal fat tissue thickness in female and male patients with procedures divided into two groups, the retroperitoneal approach (**a** and **b**) and transperitoneal approach (**c** and **d**)

Four scatter plot graphs which indicate the correlation between the operative time and retroperitoneal fat tissue thickness in female and male patient groups that underwent either retroperitoneal or transperitoneal LPN are shown in Figs. 3a, b, c and d. In female patients, the values of Pearson’s coefficient between the operative time and retroperitoneal fat tissue thickness were 0.421 (*P* = 0.029) and −0.126 (*P* = 0.595) in the retroperitoneal and transperitoneal LPN groups, respectively. In male patients, the values of Pearson’s coefficient between the operative time and retroperitoneal fat tissue thickness were 0.280 (*P* = 0.010) and 0.448 (*P* = 0.002) in the retroperitoneal and transperitoneal LPN groups, respectively.

### Linear logistic regression analyses of factors to predict the operative time of retroperitoneal and transperitoneal LPN

In retroperitoneal LPN, linear logistic regression assessment showed that the retroperitoneal fat tissue thickness (*P* = 0.005), tumor size (*P* < 0.001), and gender (*P* = 0.006) were significantly associated with operative time (Table 2). The coefficient of determination of the prediction model was 0.317.

**Table 2:** Summary of the linear regression model for variables predicting the operative time of retroperitoneal laparoscopic partial nephrectomy

A. Retrospective approach (*N* = 111)															
	Model 1					Model 2					Model 3				
Valuable	B	B SE	β	*P* value	95% COI for B	B	B SE	β	*P* value	95% COI for B	B	B SE	β	*P* value	95% COI for B
(Constant)	153.90	5.22				120.09	10.50				88.75	15.10			
Fatty tissue	1.80	0.38	0.41	<0.001	1.06–2.55	1.61	0.36	0.37	<0.001	0.89–2.32	1.11	0.39	0.26	0.005	0.34–1.89
Tumor size						1.51	0.41	0.31	<0.001	0.69–2.33	1.54	0.40	0.31	<0.001	0.75–2.33
Gender											20.60	7.33	0.25	0.006	6.07–35.13
R2				0.175					0.266					0.317	
B. Transperitoneal approach (*N* = 64)															
	Model 1														
Valuable	B	B SE	β	*P* value	95% COI for B										
(Constant)	166.14	6.90													
Fatty tissue	1.22	0.44	0.33	0.008	0.34–2.10										
R2				0.110											

*B* Unstandardized coefficients, *B SE* Standard error of unstandardized coefficients, β Standardized coefficients, *R2* Coefficient of determination

In transperitoneal LPN, retroperitoneal fat tissue thickness (*P* = 0.008) were significantly correlated with operative time (Table 2). Coefficient of determination of the prediction model was 0.110.

## Discussion

The gender of the patient has not been considered a crucial factor in deciding on the treatment strategy for small renal masses so far. However, several studies reported that there are some differences in clinical and pathological findings of renal tumors between men and women [8–10]. A few studies indicated the treatment option for renal tumors is different between men and women; for instance, women tend to undergo rather radical nephrectomy more than PN compared with men [15], although the precise reason for these gender differences had not been elucidated yet. We had a clinical impression that the operative time of LPN is longer in male patients than in female patients possibly because the management of fat tissue surrounding the kidney seems to be more difficult in male patients than in female patients. Therefore, we aimed to investigate the gender differences in operative parameters, especially operative time, in LPN. To our knowledge, the present study is the first attempt to examine this issue.

Among the operative parameters in retroperitoneal LPN, the operative time showed a gender difference. The operative time was significantly longer in male patients than in female patients. To examine the factors affecting the operative time of LPN, the prediction model for operative time of LPN was developed. According to this prediction model, the operative time of retroperitoneal LPN was proven to be predictable, and predictive factors involved the three factors of gender, maximum tumor diameter, and retroperitoneal fat tissue thickness. These results indicated that one of the possible reasons for gender discrepancy in the operative time in retroperitoneal LPN is the gender difference in retroperitoneal fat tissue thickness. Figure 3 shows that larger retroperitoneal fat tissue thickness contributed to the longer operative time in both female and male patients who underwent retroperitoneal LPN.

On the other hand, in transperitoneal LPN, there was no gender difference in the operative time, possibly because there was no positive correlation in female patients between operative time and retroperitoneal fat tissue thickness, in contrast to the procedures in male patients. In general, retroperitoneal fat tissue should neither interfere with visualization nor occupy the operative field during the transperitoneal approach, but the finding indicated that the procedure in male patients might be strongly affected by fat tissue surrounding the kidney, since the fat tissue surrounding the kidney of males seems to be harder than that of females. In addition, the present prediction model for operative time of transperitoneal LPN showed lower accuracy, which implied that the prediction of the operative time might be difficult on the basis of preoperative parameters.

Operative time influences surgical outcomes, operation-related and indirectly anesthesia-related complications, and procedural cost-effectiveness, so the precise prediction of the operative time might help to maximize the surgical outcome and reduce any perioperative complications. In fact, in the other clinical surgical fields, the analysis of the factors affecting the operative time had been conducted and a prediction model of operative time had been developed [16–19], but there is no prediction model for the operative time of LPN. The centrality-index (C-Index) [20], preoperative aspects and dimensions used for anatomic (P.A.D.U.A.) classification [21], and R.E.N.A.L. nephrometry score were developed as standardized scoring systems to quantify anatomic characteristics of kidney tumors and a few reports indicated that these three scores failed to predict the operative time although they showed correlation with warm ischemia time [22, 23].

In the past, the impact of visceral fat area on surgical outcome were well elucidated [24, 25], and some studies indicated that the larger visceral or peri-organ fat areas prolonged the operative time of laparoscopic radical nephrectomy [26], laparoscopic adrenalectomy [27], and laparoscopic radical prostatectomy [28]. In the present study, we measured the retroperitoneal fat tissue thickness using preoperative standard 2-dimensional transverse CT scan images. This retroperitoneal fat tissue thickness was significant and an independent factor for predicting the operative time of retroperitoneal and transperitoneal LPN. In fact, retroperitoneal fat tissue thickness strongly correlated with the operative time of retroperitoneal LPN in female and male patients and transperitoneal LPN in male patients. This method of measurement is simple and does not need any specialized technique and imaging device; therefore, the clinical use of this parameter might be easy.

In regard to the other operative parameters, the resected tumor volume was significantly higher in male patients in both retroperitoneal and transperitoneal LPN, and this association might be similar to that of retroperitoneal fat tissue thickness. As described in the method section, the real weight of surgical specimens including the tumor and surrounding tissue represented the resected volume in this study. This method might have led to a larger resected tumor volume because of the increased amount of retroperitoneal fat tissue.

This study has some limitations. First, it was a retrospective study, although consecutive patients were included in the analysis. Furthermore, this study involved a single surgeon and a single center. Multicenter and multinational assessments will be needed to test the reliability of our findings. Nonetheless, we believe that evaluation of the surgical outcomes of a single surgeon in a single center enabled us to exclude many confounding factors such as differences in surgical techniques.

## Conclusions

The present study is the first attempt to investigate the gender difference in the operative parameters of retroperitoneal and transperitoneal LPN. This study indicated that there is a gender difference in the operative time of retroperitoneal LPN that is possibly associated with the gender difference in the retroperitoneal fat tissue thickness. In addition, the operative time of retroperitoneal LPN significantly correlated with gender, maximum tumor diameter, and retroperitoneal fat tissue thickness determined by CT. We developed a prediction model for the operative time of retroperitoneal LPN on the basis of preoperative parameters. Interestingly, in transperitoneal LPN, the gender difference in operative time was not apparent and the prediction of the operative time might be difficult on the basis of preoperative parameters.

## Abbreviations

C index: Centrality index
CT: Computed tomography
LPN: Laparoscopic partial nephrectomy
PADUA: Preoperative Aspects and Dimensions Used for an Anatomical classification
PN: Partial nephrectomy
RCC: Renal cell carcinoma
